# Huge mediastinal ancient schwannoma causing acute respiratory failure: a case report

**DOI:** 10.1186/s13019-024-02605-1

**Published:** 2024-03-15

**Authors:** Hsuan-Ying Huang, Chien-Ming Lo, Hung-I Lu, Jen-Ping Chang

**Affiliations:** 1 Department of Pathology, Kaohsiung Chang Gung Memorial Hospital, Kaohsiung City, Taiwan, ROC; 2 Department of Thoracic and Cardiovascular Surgery, Kaohsiung Chang Gung Memorial Hospital, 123, Tapei Rd., Niaosung District, Kaohsiung City, 833 Taiwan, ROC

**Keywords:** Mediastinal schwannoma, Respiratory failure

## Abstract

Benign mediastinal tumor is usually asymptomatic and exhibits uncomplicated clinical course. Posterior mediastinal schwannoma is common, but a huge benign tumor causing acute respiratory failure due to mass effect is unusual. We present a patient who suffered from acute respiratory failure due to huge mediastinal mass effect and improved after en bloc surgical resection. A 56-year-old woman had no history of systemic disease, but experienced general discomfort and malaise for several months. She was referred to our emergency department after developing sudden respiratory failure. Intubation was performed with ventilator support and she was admitted to the intensive care unit. Chest radiograph and computed tomography showed a huge mass over the left pleural cavity causing left lung, heart, and mediastinal compression. After en bloc resection, she was weaned off the ventilator successfully and was discharged at 24 days after the operation. Postoperative outpatient follow-up showed no symptoms. Mediastinal ancient schwannoma is a rare posterior mediastinal benign tumor. However, mass effect might lead to lethal complications. En bloc resection is necessary for curative treatment.

## Introduction

Mediastinal masses span a wide histopathological and radiological spectrum [1]. Most of posterior mediastinal solid tumor is neurogenic tumors. Mediastinal schwannoma is benign in nature. Schwannoma causing complications other than tumor-related ones such as direct invasion of adjacent organs is rare. En bloc resection is the best curative treatment option. Schwannoma that has increased to a huge size causing mediastinal deviation may lead to severe complications and is hard to manage. This situation is termed as “mediastinal mass syndrome” [2]. We present a case of a patient with huge left posterior mediastinal schwannoma causing respiratory failure.

## Case report

**Fig. 1:**
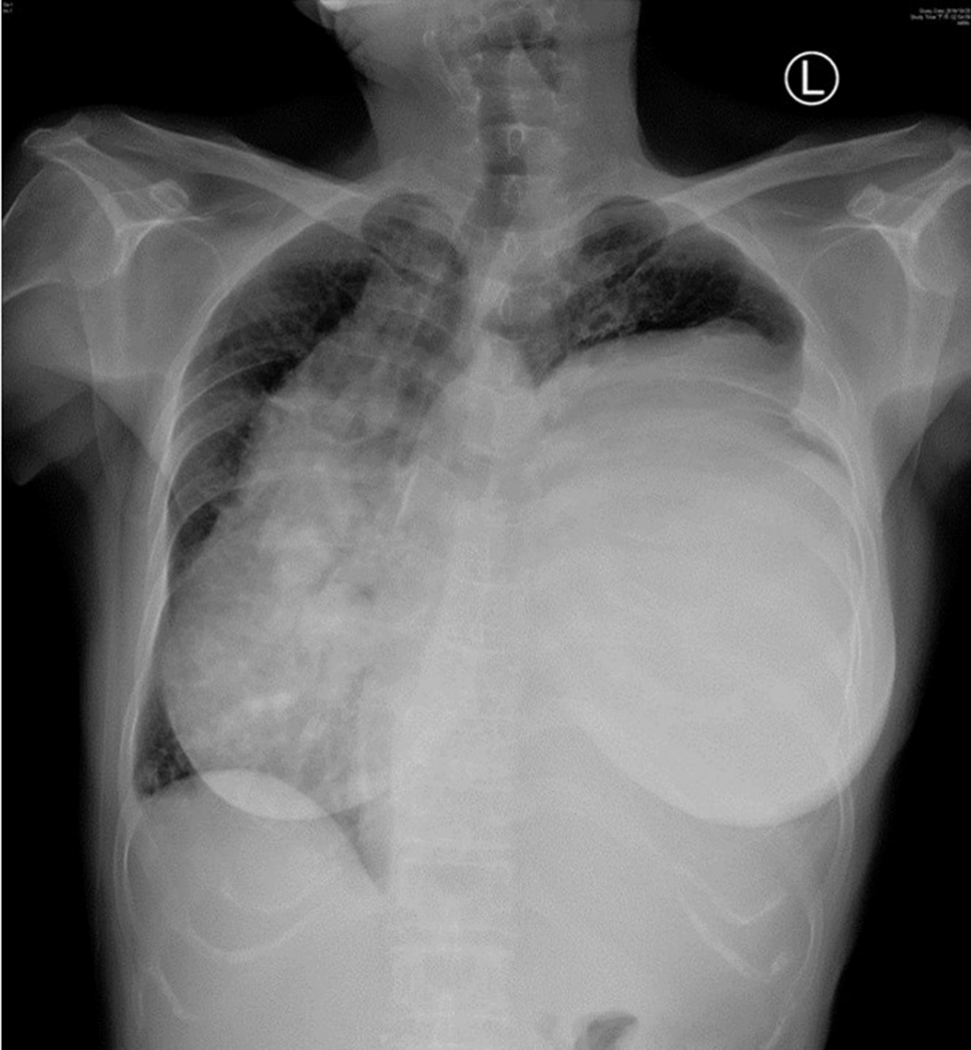
Plain radiograph of the chest

**Fig. 2:**
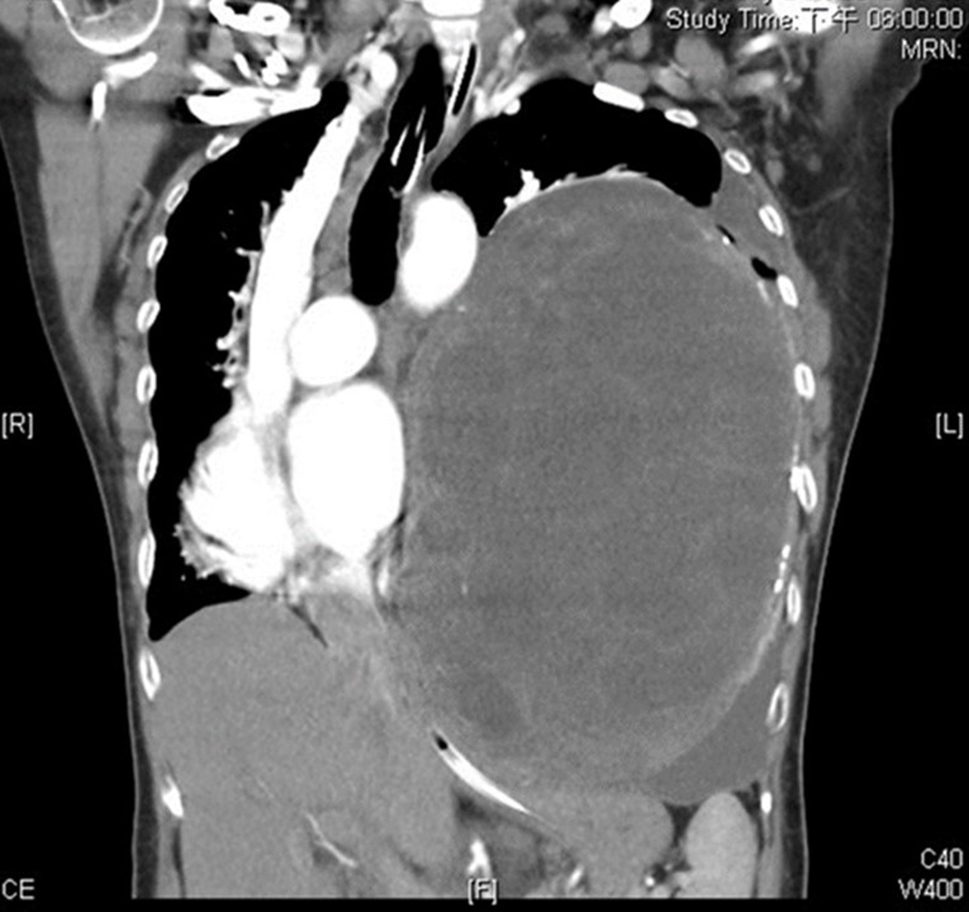
Chest computed tomography showed huge tumor causing heart compression

A 56-year-old retired woman was healthy before admission. She experienced gradual exertional dyspnea for 1 month. She had no history of other associated symptoms or signs such as cough, fever, chest pain, limb edema, oliguria, orthopnea, or paroxysmal nocturnal dyspnea. She visited our emergency department with a complaint of dyspnea. Physical examination revealed a decrease in the breathing sound on the left side. Plain radiograph of the chest revealed left lower lung consolidation and mass-like lesion over the left pleural cavity (Fig. 1). Due to impending respiratory failure, intubation was performed in the emergency department and she was admitted to the intensive care unit. Chest computed tomography (CT) with contrast enhancement was performed for detailed evaluation (Fig. 2). CT confirmed the presence of a huge pleural mass on the left side that caused deviation of the mediastinum, heart, and trachea to right side. Bronchoscopy and upper gastrointestinal panendoscopy did not reveal any endobronchial lesion or a lesion of the gastrointestinal mucosa. Surgery was performed after consulting a chest surgeon and an anesthetist.

**Fig. 3:**
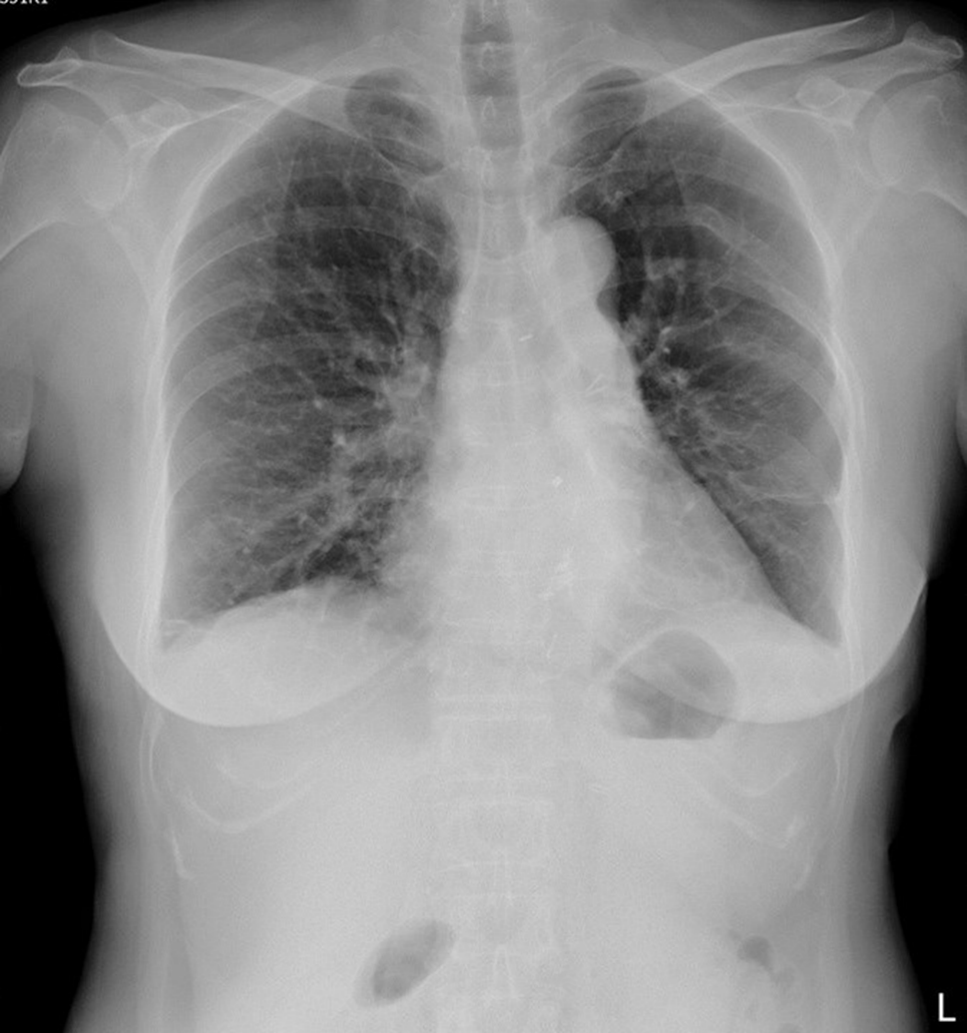
Chest radiograph at the follow-up

We used posterolateral thoracotomy via 7th intercostal space for en bloc resection of the tumor. We kept the bilateral forearm radial artery line, EKG lead, and central venous catheter as monitors. Plain radiograph of the chest after surgery showed improvement in the left lower lung consolidation. Patient was extubated at 3 days after the surgery. However, pneumonia and respiratory failure developed again on the following day. We performed intubation with ventilator support and antibiotic treatment was administered. The patient was extubated again 11 days later and was discharged 20 days later. Pathological examination confirmed ancient schwannoma. Regular outpatient follow-up with chest CT showed no recurrence (Fig. 3).

The final specimen was illustrated as Fig. 4 (Fig. 4) which size is measuring 16.5 × 12.5 × 12.0 centimeter and weighing 1358.5 g. The pathologic findings were illustrated as Figs. 5, 6, 7, 8, 9, 10, 11 and 12 (Figs. 5, 6, 7, 8, 9, 10, 11 and 12).

**Fig. 4:**
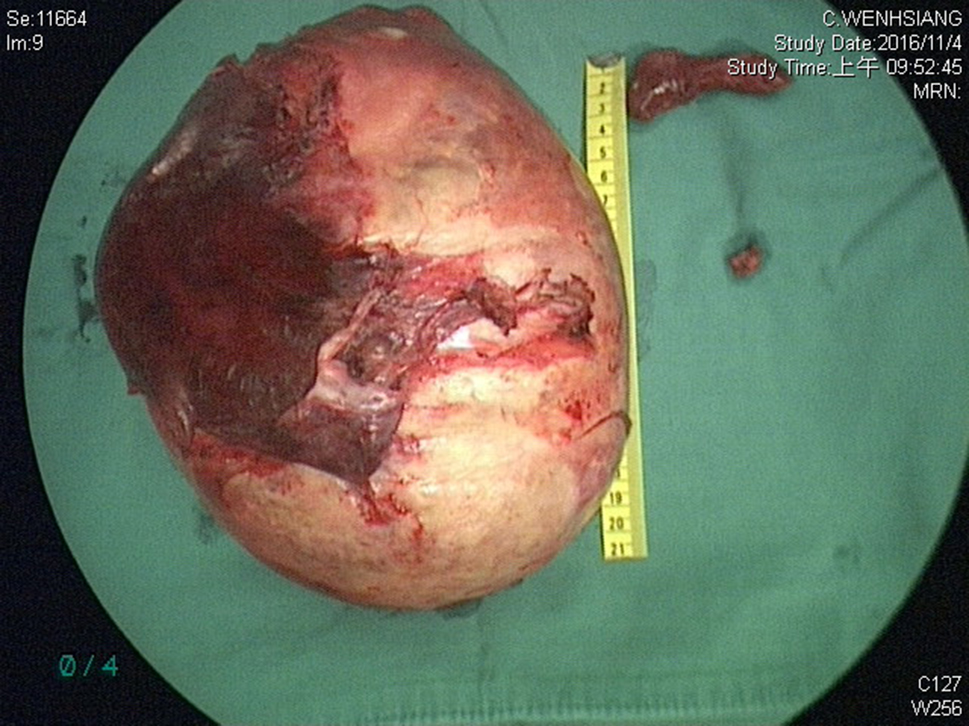
Gross picture with scale

**Fig. 5:**
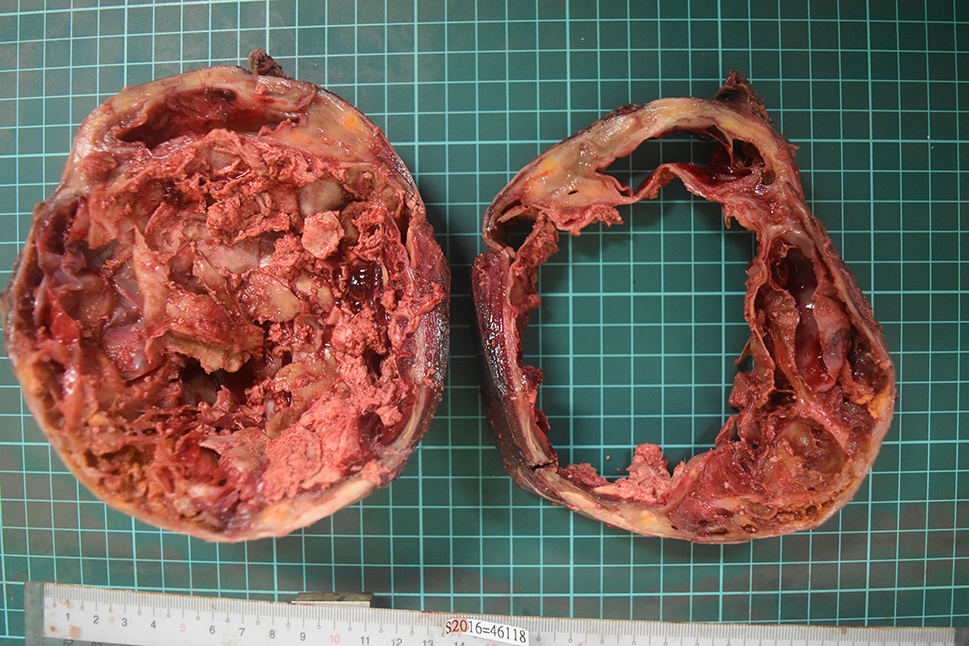
Gross picture of the tumor (cross section)

**Fig. 6:**
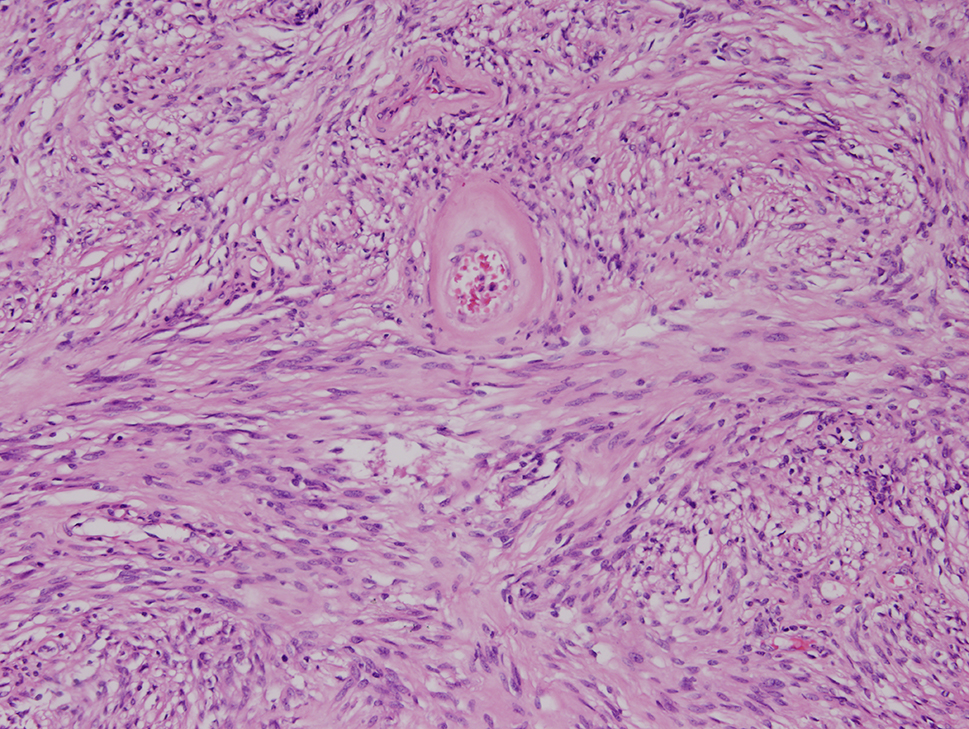
Fascicles of bland spindle to oval neoplastic cells (hematoxylin-eosin, original magnification x 100)

**Fig. 7:**
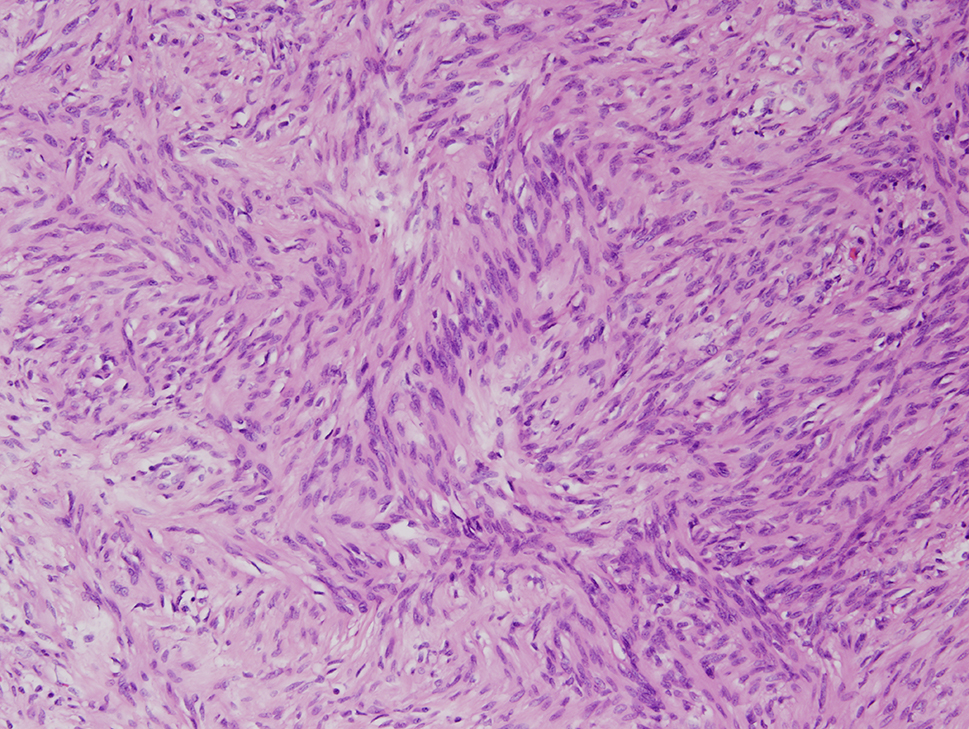
Nuclear palisading (hematoxylin-eosin, x100)

**Fig. 8:**
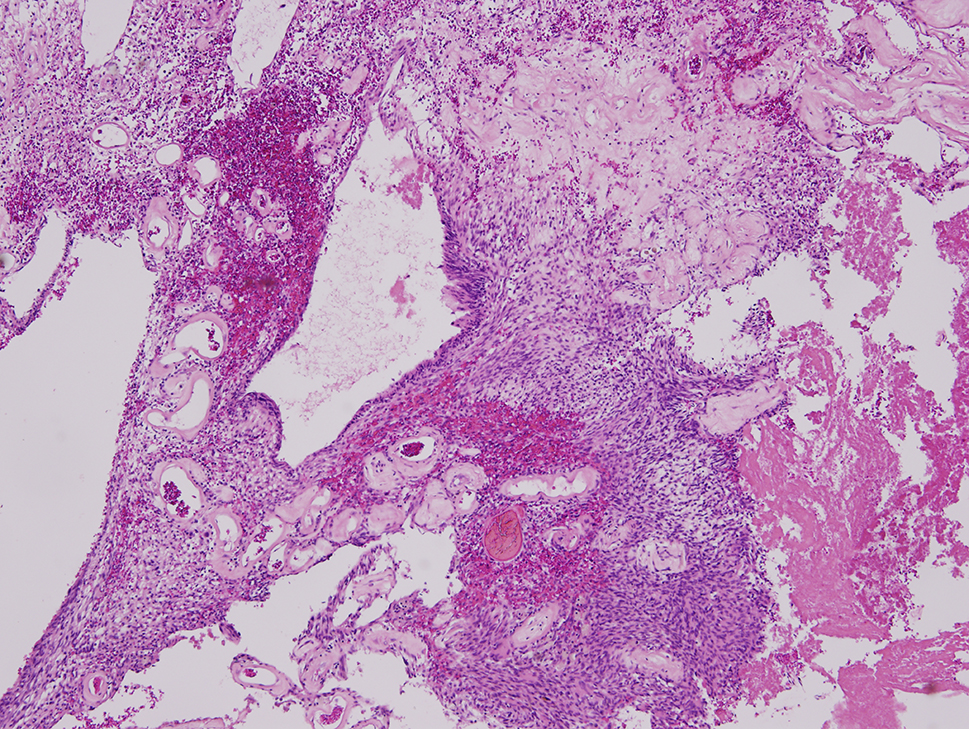
Cystic change and hyalinized vessels (hematoxylin-eosin, x40)

**Fig. 9:**
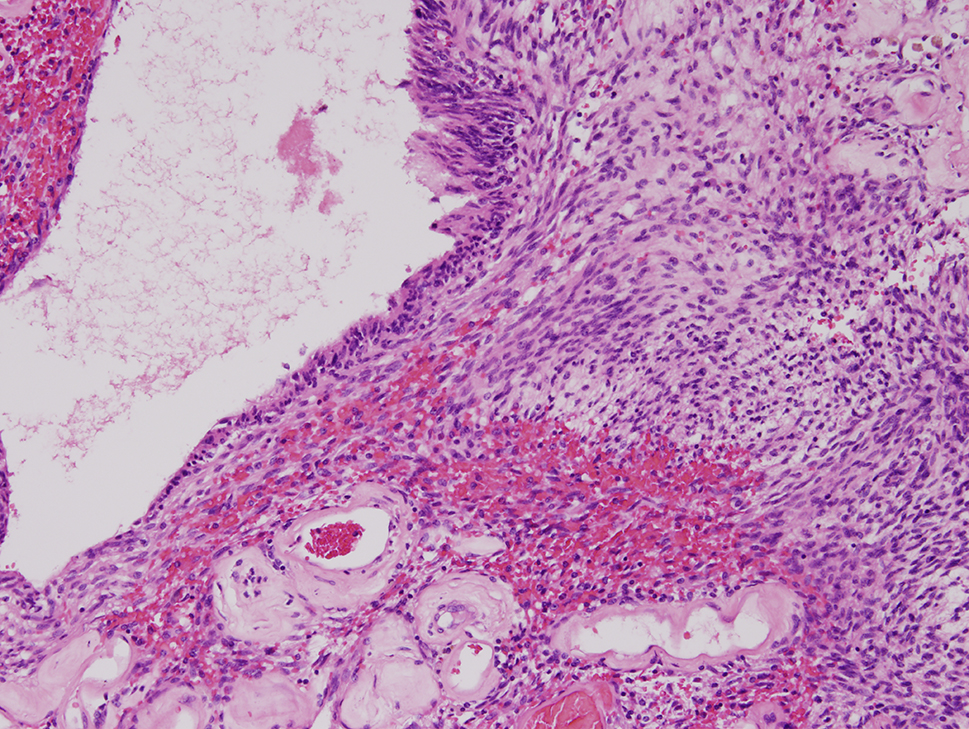
Cystic change with pseudoglandular appearance (hematoxylin-eosin, x100)

**Fig. 10:**
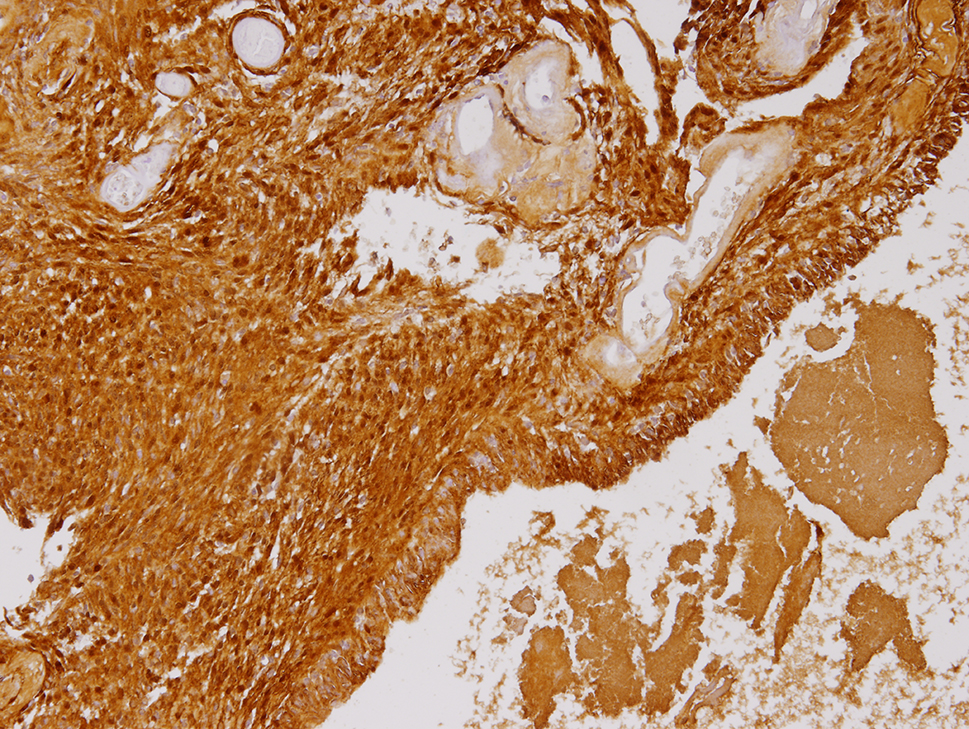
The neoplastic cells diffusely express S100 (x100)

**Fig. 11:**
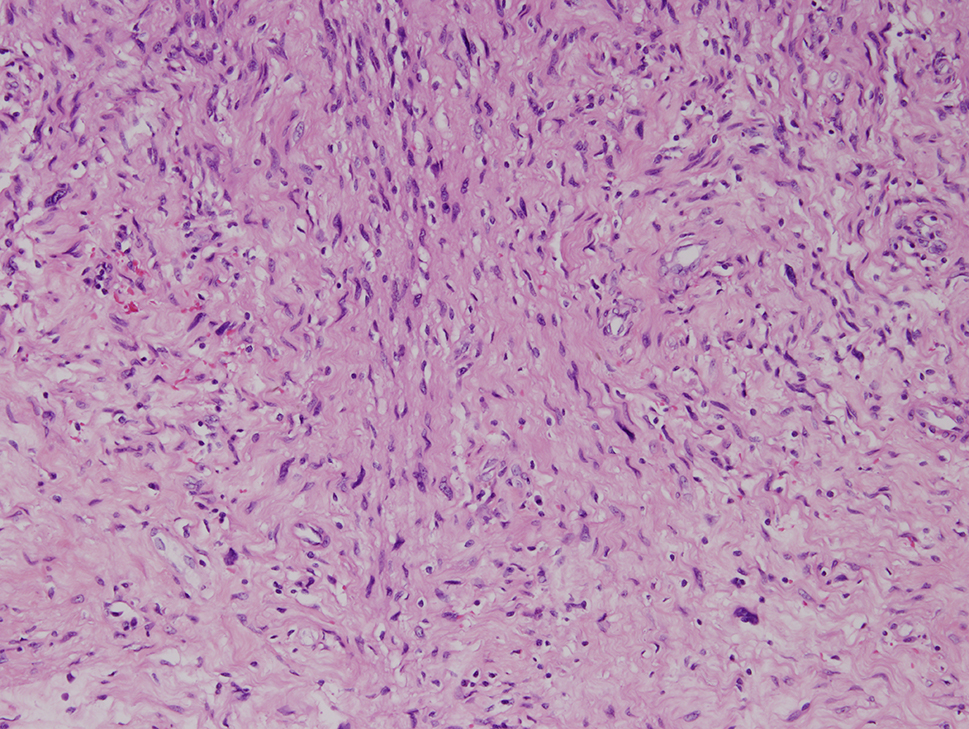
Degenerative nuclear atypia is noted focally (hematoxylin-eosin, x100)

**Fig. 12:**
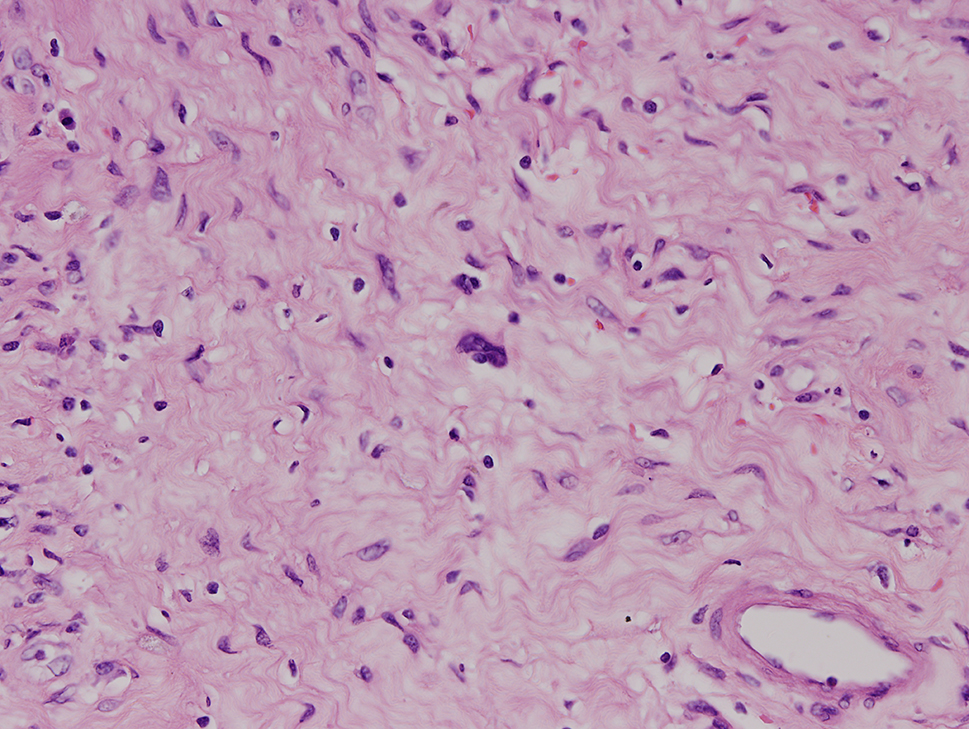
Degenerative nuclear atypia (hematoxylin-eosin, x200)

## Discussion

Mediastinal tumors span many different diagnoses and etiologies. Location in the image survey may narrow the possible diagnosis and affect the treatment [1].

A huge mediastinal mass is difficult to manage due to its anatomical location. A case report published in 2016 described a huge schwannoma over the right pleural cavity without any symptoms. En bloc resection was performed via thoracotomy, achieving good prognosis [3].

Some case series have suggested that en bloc surgical resection is the most important treatment option. Most of the huge benign mediastinal tumors exhibited good prognosis after complete resection [4]. Patients with huge benign mediastinal tumors are usually asymptomatic or exhibit minor signs such as cough, gradually developing orthopnea, and tachycardia. However, in the present case, acute respiratory failure developed due to mass effect and due to mediastinal deviation that caused compression of the trachea and the left main bronchus. It’s difficult to make differential diagnosis before operation due to large tumor burden and mass effect causing mediastinum shift. We tried panendoscope and bronchoscope to find some specific clues for diagnosis but in vain. We used thoracoscopic exploration at first and confirmed that the lesion is well-defined tumor. Thus we went on thoracotomy for en bloc resection.

Surgical management is difficult in such cases due to the poor general condition and the possibility of circulation collapse after anesthesia. Extracorporeal membrane oxygenation must be on standby for life support during the operation. One review suggests reducing the tumor size with other treatment options such as chemotherapy or radiotherapy if the pathology could be confirmed before the operation [5].

## Conclusion

Huge mediastinal schwannoma is rare, but benign in nature. En bloc surgical resection is the best treatment option for this clinical condition. A multidisciplinary approach including anesthesia physician, cardiac surgeon, and oncologic physician is desirable for better management of the patient.

## Data Availability

All data generated or analyzed during this study are included in this published article.
